# Computer Vision and Facial Landmark Detection in Dermatology: A Proof‐of‐Concept Study

**DOI:** 10.1111/exd.70233

**Published:** 2026-02-25

**Authors:** Christopher A. Guirguis, Lauren M. Ching, Joe K. Tung

**Affiliations:** ^1^ Georgetown University School of Medicine Washington DC USA; ^2^ University of Pittsburgh School of Medicine Pittsburgh Pennsylvania USA; ^3^ Department of Dermatology University of Pittsburgh Medical Center Pittsburgh Pennsylvania USA

**Keywords:** 3D facial analysis, cosmetic dermatology, smartphone‐based imaging, teledermatology

## 1 Background

Dermatologic diseases commonly involve the face with slight changes having a profound impact on patients' self‐image. While many dermatologic conditions initially appear limited to superficial layers of the skin, the associated inflammation often extends deeper [1]. In conditions such as phymatous rosacea, cystic acne, morphea, and hidradenitis suppurativa, these changes may result in thickening of the skin, hypertrophic scarring, and noticeable volumetric changes [2]. While imaging modalities exist to track changes to skin surface and texture, more profound volumetric changes in conditions such as rhinophyma may be difficult to reliably track [3]. Advances in consumer‐grade software have allowed for improved accuracy of facial landmark recognition using smartphones; public software packages in the last 2 years have allowed for three‐dimensional (3D) landmark tracking from two‐dimensional (2D) images [4]. This study explores publicly available, smartphone‐based 2D and 3D monocular facial landmark recognition packages and their accuracy in measuring fixed distances across landmarks in a young, healthy population. Specifically, we assess the accuracy of two packages in measuring alar width (AW) as a proof‐of‐concept (POC) study for application in dermatology.

## 2 Methods

**FIGURE 1:**
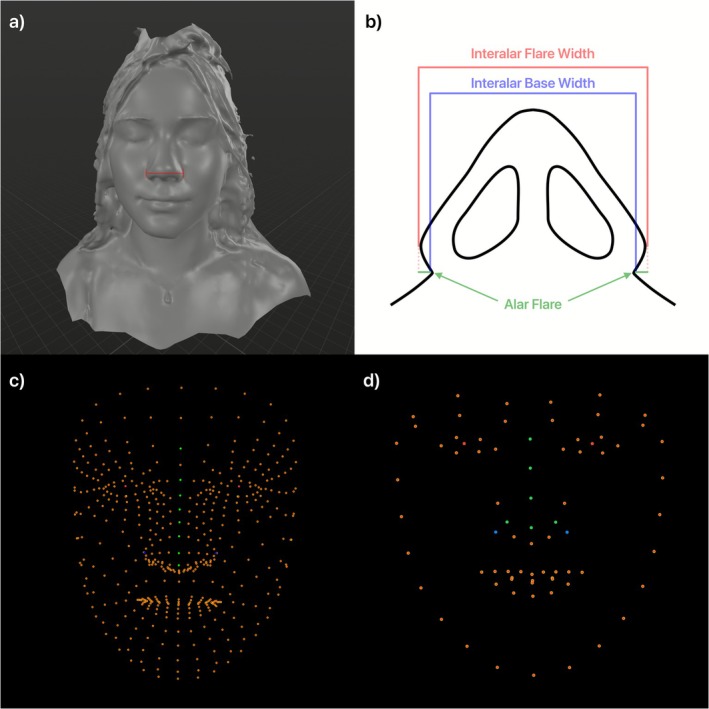
(a) A 3D model of a subject indicates the measurement used to determine AW, both in the real‐world measurements as well as using the AVK and GMP analyses. (b) The measurement taken is shown from an inferior view. The interalar flare width was used which is the summation of both the interalar base width as well as the alar flare on both sides. (c) The GMP model can identify 468 facial landmarks per frame. The red dots indicate the pupils, the green dots indicate the dorsum of the nose, and the blue dots indicate the lateralmost extents of the right and left alae which were used to measure AW. (d) The AVK model captures 76 facial landmarks per frame. The red dots indicate the pupils, the green dots indicate what is considered to be the “nasal crest” by the AVK, and the blue dots indicate the lateralmost extension of the right and left alae which were used to measure AW.

Measurement of real‐world interpupillary distance (IPD) and AW was performed with a ruler. AW was calculated using the interalar flare width (Figure 1). Computed measurements were then taken using Apple's VisionKit (AVK) and Google's MediaPipe (GMP) libraries [4, 5]. Images were captured without standardization of lighting, reflecting intended real‐world clinical use. AVK was used for preprocessing in both image capture modes to ensure pitch, roll, and yaw angles were zero radians during image capture. Landmarks captured were the bilateral pupils and lateral‐most margins of the alar border. Pixelwise AW and IPD were calculated using the distance formula and scaled to centimetres using the cm‐pixel IPD ratio and the real‐world IPD.

$${\text{Distance}}_{\text{pixels}}=\sqrt{\sum_{i=1}^{n}x_{i}^{2}}$$

$$\text{Scaling Factor}=\frac{\mathrm{IP}D_{\mathrm{cm}}}{{\mathrm{IPD}}_{\text{pixels}}}$$

$${\mathrm{AW}}_{\mathrm{cm}}={\mathrm{AW}}_{\text{pixels}}*\text{Scaling Factor}$$

AW was measured in 2D and 3D using GMP, but only 2D using AVK as AVK lacks 3D capabilities. A paired *t*‐test was run to assess statistically significant differences between the two measurement methods. Agreement between the two methods was assessed using Bland–Altman analysis with a 95% statistical limit of agreement (LOA). Informed consent was obtained by all participants.

## 3 Results

Three participants were used in this analysis, with an average real‐world IPD of 5.90 cm (SD = 0.30) and AW of 3.87 cm (SD = 0.38). The average computed AW using both the GMP 3D and 2D methods was 3.86 (SD = 0.32) with an average error of 2.36%. The average computed AW using the AVK 2D method was 4.96 (SD = 0.55) with an average error of 28.30%. Paired‐samples *t*‐test revealed no significant difference in AW using the 2D or 3D GMP estimates versus real‐world values (t (2) = 0.15, *p* = 0.89), whereas there was a significant difference when using the AVK measurement (t (2) = −6.33, *p* = 0.02). Additionally, upon Bland–Altman analysis, the mean difference was 0.01 cm with 95% LOA of ±0.22 units for GMP estimate, and −1.09 cm with 95% LOA of ±0.59 for AVK measurements.

## 4 Discussion

Monitoring of changes to facial morphology continues to be of interest in dermatology with recent literature focusing on improving reliability of systems [6]. While literature has proven the reliability of relative measurements, our data validates the reliability of absolute measurements using one known real‐world measurement, IPD. As a POC, this study prioritises methodological feasibility over statistical power, consistent with its exploratory design. The results of this study highlight the accuracy of public facial landmark recognition packages in calculating facial morphology. The difference in accuracy between GMP and AVK is likely due to the detail provided by each model, as AVK and GMP report 76 and 468 landmarks, respectively (Figure 1). The accuracy of this system can enable earlier and continuous monitoring of facial changes, allowing patients and practitioners to objectively monitor outcomes. Applications of smartphone‐based facial analysis may help bridge the gap in underserved areas as patients are able to communicate objective changes in their health remotely without added financial burden. Additionally, within cosmetic dermatology these applications may considerably decrease the cost barrier to quantitative morphologic analysis while letting patients assess landmarks beyond alar width, including philtrum height, lip height, and upper‐ to lower‐lip ratios using the 468 landmarks assessed in the GMP analysis [4, 6]. These facial measurements can enhance cosmetic consultations for patients through objective measures before treatments such as filler injections, buccal lipectomy, or cheek liposuction thereby giving patients a quantitative means for tracking outcomes. The primary limitation of this study is the sample size, as a more heterogeneous cohort may strengthen external validity. Future studies should focus on analysis of other facial measurements and incorporation of Light Detection and Ranging (LiDAR) to augment measurements.

## Author Contributions

Conceptualization: C.A.G., J.K.T. Data curation: C.A.G., L.M.C. Formal analysis: C.A.G.; Supervision: J.K.T. Writing – original draft preparation: C.A.G., L.M.C. Writing – reviewing and editing: L.M.C., C.A.G. All authors have read and approved the final manuscript.

## Funding

The authors have nothing to report.

## Ethics Statement

All participants provided informed consent, and data confidentiality was maintained throughout the study.

## Consent

Consent obtained from participants.

## Conflicts of Interest

The authors declare no conflicts of interest.

## Data Availability

The data that support the findings of this study are available on request from the corresponding author. The data are not publicly available due to privacy or ethical restrictions.
